# Temporal Interactions between Neural Proxies forMemory Recall, Negative Affect, and EmotionRegulation in Major Depression

**DOI:** 10.21203/rs.3.rs-4298308/v1

**Published:** 2024-05-30

**Authors:** Christina Michel, Mike Schmidt, J. John Mann, Sarah Herzog, Kevin Ochsner, Lila Davachi, Noam Schneck

**Affiliations:** Columbia University Irving Medical Center; Columbia University Irving Medical Center; Columbia University; Columbia University; Columbia University Irving Medical Center; Columbia University Irving Medical Center; Columbia University Irving Medical Center

## Abstract

Dysfunction in emotion regulation (ER) and autobiographical memory are components of major depressive disorder (MDD). However, little is known about how they mechanistically interact with mood disturbances in real time. Using machine learning-based neural signatures, we can quantify negative affect (NA), ER, and memory continuously to evaluate how these processes dynamically interact in MDD. Unmedicated individuals with MDD (*N*=45) and healthy volunteers (HV; *N*=38) completed a negative autobiographical memory functional magnetic resonance imaging task wherein they recalled, distanced from (an ER strategy), and immersed into memories. We used a negative affect signature (PINES) and an emotion regulation signature (ERS) to quantify moment-to-moment NA and ER. We then examined whether memory engagement, indexed by hippocampal activity, predicted subsequent change in PINES and ERS over time. During memory recall and immersion, greater hippocampal activity predicted increased PINES across groups. During distancing, greater hippocampal activity in HVs predicted increased ERS but not PINES. In MDD, greater hippocampal activity predicted increased PINES but not ERS. Findings suggest abnormalities in the real-time relationship between memory, NA, and ER in MDD. During distancing, as predicted, HVs showed an attenuation of the linkage between memory engagement and NA, and they had subsequent increases in ER following memory reactivation. In contrast, MDD was characterized by continued linkage between memory engagement and NA, without subsequent increases in ER. Deficits in engagement of ER and ineffective modulation of NA following negative memory recall may contribute to the mood disturbances in MDD and are potential targets for clinical intervention.

## INTRODUCTION

Major depressive disorder (MDD) is a leading cause of disability worldwide^1^. Impairments in emotion regulation and biases in memory processes are core facets underlying MDD^2–7^. While there is extensive research documenting these impairments in depressed individuals, the neural basis for these processes, as well as how they mechanistically contribute to depressive symptoms, is not well understood.

Autobiographical memories represent a critical area of dysfunction in MDD^2, 7^. How individuals interpret, process, and recall personal events impacts their thoughts about themselves and their future^2, 8^. For example, it has been shown that individuals with MDD have a bias to both encode and recall negative memories^2, 9^. Depressed individuals recall negative memories faster and more frequently compared with positive memories^2, 9^. Compared with never-depressed individuals, those with a history of depression also have impoverished recall for positive events: positive events are less vivid, less specific, and provide less benefit emotionally^2, 9–13^. Additionally, depressed individuals exhibit dysfunctional rumination and avoidance patterns with negative memories^2, 14^. The ability to effectively regulate one’s emotional response to negative events and memories is critical for maintained well-being^2, 4^. Therefore, it is clear that dysfunction in memory processes, coupled with impairments in emotion regulation, contribute to the cognitive and mood symptoms of MDD^2, 7, 9^.

Impaired emotion regulation is also well documented in depression^4–6^. Depressed individuals employ more ineffective emotion regulation strategies (e.g., suppression, avoidance) and underuse effective strategies, like cognitive reappraisal and acceptance^4–6^. Additionally, lab-based studies have shown that neural processes underlying emotion regulation, which involves both higher level cognitive structures and subcortical limbic structures, differ in MDD compared with healthy volunteers for negative stimuli^15^. This work has largely been conducted using standardized stimulus sets such as sad faces, negatively-valanced words, and upsetting scenes^15^. However, differences in the neural mechanisms of negative affect and emotion regulation during more personalized or clinically relevant stimuli, like personal negative autobiographical memories, are less well understood.

Neural processes for regulating negative affect in response to upsetting memories in individuals with MDD appear to differ from that of healthy volunteers (HVs)^16^. Prior work found increased amygdala-hippocampal connectivity mediated the relationship between higher self-report negative affect ratings in depressed subjects compared with HVs^16^, suggesting that processes supported by the hippocampus may contribute to the negative affect experienced while recalling negative autobiographical memories. Additionally, depressed subjects showed a larger reduction in posterior hippocampal activation compared to HVs during reappraisal of autobiographical memories^16^, indicating that reductions in hippocampal activity may be related to an emotion regulation strategy employed specifically by depressed individuals^16^. When done correctly, cognitive reappraisal involves adjusting one’s emotional reaction to a negative memory without having to suppress the memory^2, 5, 17, 18^. However, because of the relationship between memory engagement and negative affect^16^, individuals with MDD may have difficulty reappraising, and may suppress the memory itself rather than reappraising their emotional response to the memory. While these findings provide initial support for neural differences in emotion regulation and negative affect during negative memories, a major limitation of prior work is that it has relied on static measures for assessing these complex psychological processes. The use of static measures (i.e., a single self-report rating, a single beta weight representing 10–20 second of task) does not allow examination of the dynamics of the relationship between memory, negative affect, and emotion regulation. In order to understand how memory engagement impacts these processes, we need to examine the temporal relationship between memory, negative affect, and emotion regulation.

Advances in analysis techniques, as well as scanner acquisition parameter options, e.g., shorter pulse sequence repetition time (TRs), have allowed for the examination of neural proxies of psychological processes, like negative affect, memory, and emotion regulation, at second-to-second intervals. Machine learning has allowed for identification of sensitive and specific neural signatures for complex psychological processes like negative affect and cognitive reappraisal^19–21^. Unlike traditional univariate approaches that examine overall magnitude of BOLD activity within a set of voxels or clusters, machine learning-based approaches identify a spatially distributed multivoxel pattern as a proxy for a given mental process^21^. Neural signatures have also been shown to be a more robust predictor of psychological processes compared with region of interest (ROI) brain regions or resting-state networks (e.g., salience network, default mode network)^19^. The Picture-Induced Negative Affect Signature (PINES) and the Emotion Regulation Signature (ERS) are validated neural signatures that serve as a proxy for negative emotion and emotional reappraisal^19, 20^. The PINES and ERS consist of weighted maps distributed across cortical and subcortical regions that can be applied to functional magnetic resonance imaging (fMRI) data to produce a value that represents the degree to which someone is engaging in negative affect or emotion regulation at each TR. Using these values, we can examine fluctuations in these psychological processes at the moment-to-moment level.

**Figure 1:**
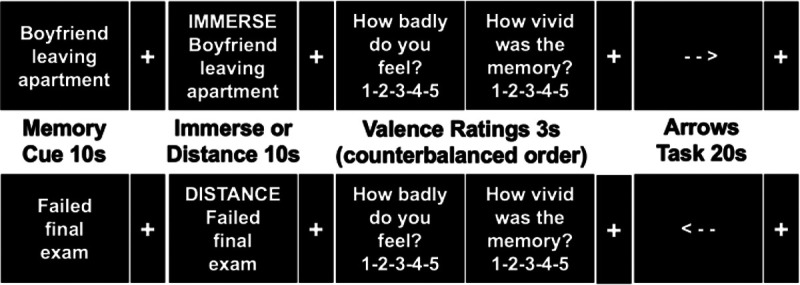
Negative Autobiographical Memories Task Note: s=seconds. Each trial begins with a memory cue for 10s that prompted participants to recall the memory. After an ISI ~2s, the memory cue is presented with an instructional cue (‘immerse’ or ‘distance’) for 10s, during which time participants either immersed or distanced themselves from their memory. Each presentation is followed by an arrows task where participants indicate the direction of the arrow. There are eight memories and four runs total. During each run, participants are presented with two memories twice, once with the immerse instruction and once with the distance instruction. Each presentation is referred to as a period, with 4 periods per run.

The current study examined whether hippocampal activity during a negative autobiographical memory fMRI task predicted subsequent negative affect measured by PINES for unmedicated depressed individuals and healthy volunteers. We also examined whether depressed individuals differed from HVs in their neural approaches to downregulating negative affect during this task. During the fMRI task, participants were asked to recall the negative memory and then instructed to immerse themselves in it or distance themselves from the negative memory (Fig. 1). We hypothesized that hippocampal activity would predict subsequent change in negative affect signature expression for MDDs and HVs during the memory cue and immerse conditions, with greater hippocampal activity predicting increased negative affect signature expression. During the distance condition, we hypothesized that there would be an interaction of group by hippocampal activity, such that greater hippocampal activity would predict subsequent increased negative affect signature expression for depressed individuals but not for healthy volunteers. We also explored differences in reappraisal strategies. During the distance condition, we hypothesized that there would be an interaction of group by hippocampal activity, such that greater hippocampal activity would predict subsequent increases in emotion regulation as measured by ERS for HVs, but not for individuals with MDD. Additionally, we examined whether reduction in hippocampal activity was used as a regulatory strategy by depressed individuals. We predicted that MDD participants would show a greater reduction in hippocampal activity when instructed to distance from the negative memory compared with HVs.

## METHODS AND MATERIALS

### Clinical Assessment

Psychiatric diagnoses were determined using the Structured Clinical Interview for DSM-IV or DSM-V by doctoral- or masters’-level psychologists^22^.

### Sample

Participants consisted of healthy volunteers (N = 38) and unmedicated individuals diagnosed with MDD (N = 45). Subjects were recruited by the Molecular Imaging and Neuropathology Division (MIND) at the New York State Psychiatric Institute (NYSPI) and Columbia Psychiatry to participate in a larger multimodal study examining depression and suicidality. All subjects were 18–65 years of age, English speaking, had normal or corrected-to-normal vision, and had no conditions that contraindicated MRI.

Depressed subjects met criteria for a current major depressive episode, were between ages 18–65 years, and were medication-free for ≥ 21 days at the time of scan. The medication washout protocol involved a one-week medication taper and three weeks off any medication that affects relevant brain systems. Exclusion criteria included: 1) lifetime psychosis; 2) substance/alcohol abuse (past 2 months), or past-year substance/alcohol dependence; 3) past-year anorexia nervosa or bulimia nervosa; 4) lifetime intravenous (IV) drug use; 5) greater than 3 lifetime incidents of 3,4-methylenedioxy-methamphetamine (MDMA) use; 6) first-degree family member with schizophrenia (for subjects under age 33); 7) significant active physical illness; 8) electroconvulsive therapy in the past 6 months (ECT); 9) previous head trauma with loss of consciousness or cognitive impairment. Healthy volunteers were eligible if they had no active medical illness, no lifetime history of Axis I or Axis II psychiatric illness, no first- or second-degree relatives with a history of a major depressive episode and were never prescribed psychiatric medications. Study procedures were approved by the institutional review board at the New York State Psychiatric Institute.

### Autobiographical Memory Collection

In a pre-scanning testing session, a clinician asked participants to recall 8 upsetting memories from the last 6 months of their lives that made them feel sad, angry, or upset. If participants had difficulty, they were told that upsetting situations with family, friends and work are often sources of distress for people and if necessary, were asked to recall memories involving feeling ashamed, humiliated, rejected, misunderstood or hopeless. Participants rated each memory on a scale of 1–10 in terms of how initially distressing it was and its current intensity and vividness (all task memories were rated as a 7 or higher). The clinician and participant created brief phrases to be used as memory cues for the fMRI task. Participants provided 4 neutral memories for training purposes.

### Autobiographical Memory Task Training

During the ‘immerse’ condition, participants were told to see the situation in the first person and to feel any emotions that may arise. During the ‘distance’ condition, participants were told to watch their memory unfold as if from a distance and to adopt the perspective of a reporter who is focused on the facts of their memory rather than its emotional details. Participants practiced the strategies with neutral memories, so they did not habituate to the upsetting memories. Participants practiced distancing and immersing two memories aloud with an experimenter before practicing silently with two additional memories. All participants successfully described the strategy to the experimenter and verbalized how to distance themselves.

### Autobiographical Memory fMRI Task

Participants completed four fMRI task runs, each comprised of four trials (Fig. 1). Each trial began with a memory cue lasting 10 seconds that prompted participants to recall the memory indicated. After a brief delay, the memory cue was re-presented with an instructional cue (‘immerse’ or ‘distance’) for 10 seconds, during which time participants either immersed or distanced themselves from their memory. There were eight memories and four runs total. During each run, each memory was recalled twice, once before each of the two conditions (i.e., immerse and distance), in counterbalanced order across subjects. Each presentation is referred to as a period, with 4 periods per run. After each distance and immerse period, subjects responded to two questions, “How badly do you feel?” and “How vivid was the memory?”, presented in a counter-balanced order over trials, on a 5-point scale, with higher ratings indicating more negative affect and memory vividness. Between trials, participants completed an active baseline task involving making button presses to indicate the direction of an arrow for 20 seconds^23^. Stimuli were presented using E-prime software (Psychology Software Tools, Inc.) on a PC computer, using an LCD projector and a back-projection screen. Participants responded using a five-finger-button-response (Avotec Inc. and Resonance Technologies).

### fMRI Acquisition

This study used anatomical and functional MRI data acquired on a 3T GE SIGNA Premiere scanner with a 48-channel head coil with sequences based on the ABCD protocols (https://abcdstudy.org/). T1-weighted (T1w) images were acquired with a 256×256 matrix at 1.0mm isotropic resolution, 2.5s TR, 2ms TE, 1.06s TI, and an 8-degree flip angle. T2-weighted (T2w) images were acquired with a 300×300 matrix at 0.8mm isotropic resolution, 3.2s TR, 60ms TE, and a 90-degree flip angle. T1w and T2w images were provided to FreeSurfer for segmentation and creation of regional masks. Structural images were used in fMRIPrep as a subject-specific space for registering BOLD data, and as an intermediate before registering BOLD data to an MNI atlas for group analyses. Functional images were acquired with a 90×90 matrix at 2.4mm isotropic resolution, 4x multiband, 0.9s TR, 26ms TE, and a 52-degree flip angle. The functional sequence was modified slightly from ABCD (from 6x to 4x multi-band) to improve signal-to-noise in frontal regions. Field maps for correcting nonuniformities in the B0 field in functional images were acquired as two phase-encoded polarity (PEPolar) spin echo EPI scans, one with forward and one with reverse polarity.

### Preprocessing & Segmentation

Pre-processing of functional images was done with fMRIPrep 20.2.0^24, 25^, without slice-timing correction due to the short TR length in these data. fMRIPrep normalized, skull-stripped, and segmented the anatomical data. It then skull-stripped the functional data and used PEPolar field maps to correct for inhomogeneity. fMRIPrep performed motion correction, and saved 4D motion-corrected images separately in native space, T1w space, and MNI152NLin2009cAsym space. Head motion estimates for each frame were saved to a confounds table, used later for further cleaning of the functional data. Reports from fMRIPrep were used to manually check the quality of pre-processing. After preprocessing we used Nilearn to apply 5mm smoothing, high and low pass filtering, adjust for motion confounds, and standardize (z-score) the BOLD signal.

Independently of fMRIPrep’s FreeSurfer processing, FreeSurfer 7.1.1 was run on both the T1w and T2w images for the participants with both modalities. For participants who did not get a T2w scan (N = 10), FreeSurfer was run on the T1w scan alone. FreeSurfer7 uses a validated Bayesian algorithm to segment the hippocampus by using the T1-weighted image and then incorporating each subjects T2-weighted image to further refine hippocampal boundaries and segmentation. Following FreeSurfer’s recon-all process, additional scripts were run to further segment hippocampal masks^26^. Masks were generated from these in original high-resolution and then resampled to each participant’s native BOLD space to allow extraction of localized BOLD signal from functional images. Then using these resampled hippocampal masks, time series data were extracted such that subjects had a value of hippocampal activity for each TR of the scan. These values served as a proxy for memory engagement.

### Negative Affect Signature (PINES)

The negative affect signature (PINES) is a whole-brain pattern of weights trained to predict negative emotion. It was trained to predict self-reported negative affect along a continuous 1–5 scale after viewing images, and reliably did so while showing specificity to negative affect as opposed to physical pain^19^. A full description of the methods can be found in the original paper^19^. In this study, each volume of cleaned BOLD activity was multiplied by PINES weights to generate a scalar value used to quantify negative affect at each time point.

### Emotion Regulation Signature (ERS)

The emotion regulation signature (ERS) is a pattern of weights trained to predict emotion regulation^20^. It was trained in an independent data set to discriminate trials where participants were asked to reappraise a negative image from trials where they were asked to simply look at a negative image. Training was performed with elastic net regression using FaSTGLZ^27^, which does automatic hyperparameter tuning to optimize performance. In contrast to PINES, the ERS was trained on voxels within a ventrofrontal region believed a priori to be broadly applicable to emotion regulation as opposed to image-specific regulation. More details can be found in the Supplemental Materials.

### Statistical Analyses

#### Behavioral Analysis

To examine both group (MDD, HV) and condition (distance, immerse) differences in the self-reported vividness and negative affect ratings, ratings were examined in separate 2 group × 2 condition mixed linear models.

To examine whether negative affect measured by self-report was associated with negative affect measured by PINES, we used mixed effects models to test for a main effect of self-report ratings on PINES. Covariates included run number (1–4), period (0–3), group, and condition. Using the same covariates, we also explored whether self-reported vividness was related to hippocampal activity using a mixed effects model testing for main effect of vividness ratings on hippocampal activity.

#### Temporal Relationship between Hippocampal Activity and Subsequent Negative Affect Signature (PINES) Expression

To examine whether hippocampal activity at one time point predicted a subsequent change in negative affect as measured by PINES, mixed effects models were used to test for a main effect of hippocampal activity at each TR on PINES 1 TR (.9 seconds) later. The same model was also run test for a main effect of hippocampal activity on PINES 2 TRs (1.8 seconds) later. Covariates included run number (1–4), period (0–3), PINES, condition (memory cue, immerse, and distance), and group (MDD, HV). We also examined whether there was an interaction of condition × group × hippocampal activity in predicting 1-TR-lagged PINES and 2-TR-lagged PINES. The initial model included all conditions, but post-hoc analyses examining group × hippocampal activity interactions were conducted independently for memory cue, immerse, and distance conditions.

#### Temporal Relationship between Hippocampal Activity and Subsequent Emotion Regulation Signature (ERS) Expression

To determine whether HVs were using compensatory strategies of cognitive reappraisal during distancing, we examined whether hippocampal activity at one time point predicted a subsequent change in emotion regulation as measured by ERS. Separate mixed effects models were used to test for a main effect of hippocampal activity on ERS at 1 TR later and 2 TRs later, respectively. In two separate models, we examined the interaction of group × hippocampal activity × condition in predicting 1-TR-lagged ERS and 2-TR-lagged ERS. Covariates included run number (1–4), period (0–3), ERS, average BOLD signal within the ERS mask, condition, and group. The initial models included all conditions, but posthoc analyses examining group × hippocampal activity interactions were conducted independently for memory cue, immerse, and distance conditions.

#### Change in Hippocampal Activity from Memory Recall to Distance Condition

To examine group differences in the change in hippocampal activity change when instructed to distance following memory recall, we used a mixed effects model to test for an interaction of group (MDD, HV) and condition (memory recall, distance) on hippocampal activity. Covariates included run number (1–4) and period (0–3).

## RESULTS

### Behavioral Results

**Figure 2:**
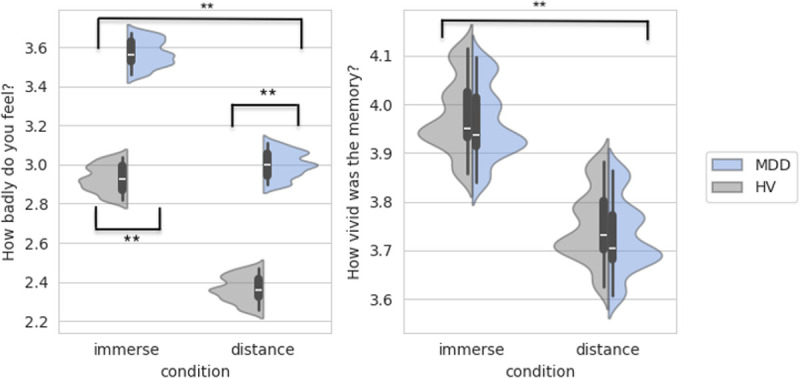
Self-Report Ratings for Negative Affect and Vividness Note: **p<.001. MDD: Major Depressive Disorder. HV: Healthy Volunteers. Higher values for negative affect indicate feeling worse. Higher values for vividness indicate more vivid memories. Negative affect self-report ratings were higher during the immerse condition than the distance condition (*p*<.001). MDDs had higher self-reported negative affect during both the immerse and distance conditions compared with HVs (*p*<.001). Self-reported vividness was higher during the immerse condition compared with distance condition (*p*<.001). There were no group differences in self-reported vividness ratings.

There were no group differences in demographic characteristics (Table 1). There was a main effect of condition on negative affect self-report ratings, such that the immerse condition had higher negative affect ratings than the distance condition for both groups (*F*_1,1130_ = 135.35, *p* < .001) (Fig. 2). There was also a main effect of group, such that the MDD group had higher negative affect during both the immerse and distance conditions compared with HVs (*F*_1,85_ = 12.93, *p* < .001) (Fig. 2). Vividness was higher during immerse compared to distance trials to a comparable degree in both groups (*F*_1,1153_ = 37.56, *p* < .001) (Fig. 2).

### Self-Report Ratings of Negative Affect and Vividness

Self-reported vividness ratings were positively correlated with hippocampal activity (*F*_4,5879_ = 2.59, *p* = .035), such that increased hippocampal activity was related to higher self-reported memory vividness. We also validated the PINES in our dataset by showing that PINES output was positively related to self-reported “feels badly” ratings (*F*_4,9113_ = 4.87, *p* < .001), with greater negative affect, i.e., higher PINES output, associated with feeling emotionally worse.

### Temporal Relationship between Hippocampal Activity and Subsequent PINES Expression

Hippocampal activity predicted change in PINES 1 TR later (*F*_1,26473_ = 18.1, *p* < .001) (Table S1), but there was no interaction of condition × group × hippocampal activity (Table S2).

**Figure 3:**
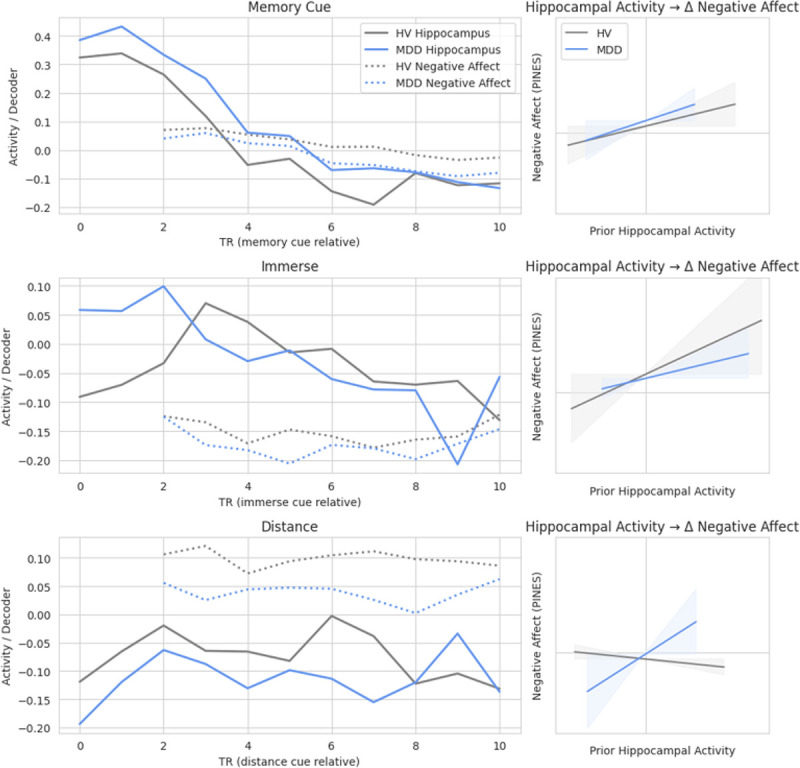
Temporal Relationship between Hippocampal Activity and Subsequent Negative Affect Signature (PINES) Expression Note: MDD: Major Depressive Disorder. HV: Healthy Volunteers. During the memory cue and immerse conditions, greater hippocampal activity predicted subsequent increases in negative affect signature (PINES) expression 2 TRs later (p<.001) for both depressed individuals and HVs. During the distance condition, there was an interaction of group by hippocampal activity (p<.001), such that greater hippocampal activity predicted subsequent increases in negative affect signature expression for MDDs, but not for HVs. Confidence intervals in the figure were approximated from simplified models due to random subject effects in full model.

Hippocampal activity also predicted subsequent change in PINES 2 TRs later (*F*_1,23826_ = 17.35, *p* < .001) (Table S3), and a three-way interaction of condition × group × hippocampal activity on change in PINES was significant (*F*_2,23813_ = 4.38, *p* = .012) (Table S4). Post-hoc analyses revealed that greater hippocampal activity predicted subsequent increases in PINES during memory recall (*F*_1,11896_ = 10.71, *p* = .001) and during immerse periods (*F*_1,6613_ = 6.52, *p* = .011) for both the MDD and HV groups (Fig. 3). During the distance condition, there was an interaction of group and hippocampal activity (*F*_1,6595_ = 5.7, *p* = .017) such that greater hippocampal activity predicted subsequent increases in PINES for participants with MDD, but not HVs (Fig. 3, Table S5).

### Temporal Relationship between Hippocampal Activity and Subsequent ERS Expression

Hippocampal activity did not predict change in ERS 2 TRs later (Table S6). There was a three-way interaction of condition × group × hippocampal activity on 2-TR-lagged ERS (*F*_2,23738_ = 7.41, *p* < .001) (Table S7). Post-hoc analyses showed that during distancing, there was an interaction of hippocampal activity and group in predicting subsequent change in ERS (*F*_1,6526_ = 16.82, *p* < .001) (Table S8). For HVs, greater hippocampal activity was associated with subsequent increases in ERS i.e., higher emotion regulation signature expression. In contrast, in the MDD participant group, hippocampal activity was negatively associated with ERS, such that more hippocampal activity predicted less emotion regulation signature expression 2 TRs later. Hippocampal activity was not related to subsequent ERS during the memory cue or immerse conditions.

There was no main effect of hippocampal activity on change in ERS 1 TR later and no interaction of condition × group × hippocampal activity on subsequent change in ERS 1 TR later.

### Change in Hippocampal Activity from Memory Recall to Distance Condition

**Figure 5:**
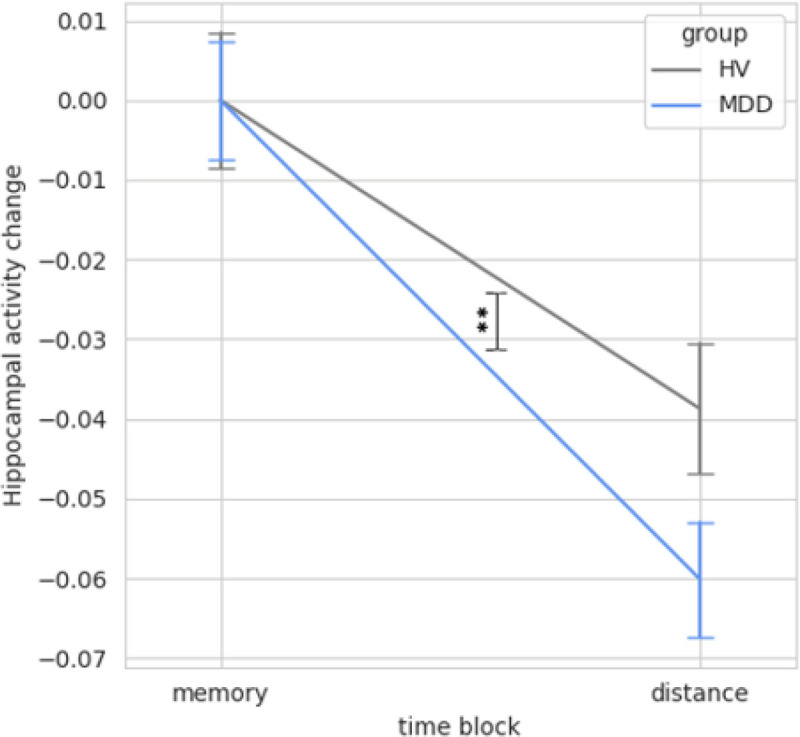
Greater Reduction in Hippocampal Activity when Instructed to Distance following Memory Recall in Depressed Individuals vs. Healthy Volunteers Note: **There was a larger decrease in hippocampal activity (p<.001) from memory recall to the distance condition for MDDs than HVs. MDD: Major Depressive Disorder. HV: Healthy Volunteers. Average hippocampal activity during the memory cue period has been set to zero for both groups to demonstrate the change in hippocampal activity from memory recall compared to the distance condition.

When transitioning from the memory recall condition to the distance condition, there was an interaction of group and condition on hippocampal activity (*F*_1,21755_ = 12.96, *p* < .001) (Fig. 5; Table S9). This interaction was caused by individuals with MDD showing a greater reduction in hippocampal activity compared to HVs.

## DISCUSSION

This is the first study to report how dynamic changes in memory engagement, indexed by hippocampal activity, during a negative autobiographical memory paradigm predicted subsequent change in negative affect signature expression and emotion regulation signature expression at a moment-to-moment level. We also demonstrated how these real-time interactions differed during reappraisal in individuals with MDD compared with healthy volunteers. During the memory recall and immerse conditions, greater hippocampal activity predicted increased negative affect signature expression for both depressed individuals and HVs, demonstrating a linkage between memory retrieval and subsequent negative affect. When instructed to downregulate their emotional response to the memory during the distance condition, HVs no longer showed a linkage between memory and negative affect signature expression and instead greater memory engagement predicted a subsequent increase in emotion regulation signature expression. In contrast, MDDs continued to show a linkage between memory engagement and subsequent negative affect signature expression, with no linkage to subsequent emotion regulation signature expression. Additionally, when instructed to downregulate their emotional response, depressed individuals showed a greater reduction in hippocampal activity compared with HVs, suggesting MDDs may disengage with negative memories as a strategy for modulating negative emotions. Taken together, these findings suggest that HVs approach regulation by weakening the automatic linkage between negative autobiographical memories and the subsequent negative affect the memory evokes, likely through cognitive reappraisal. By contrast, MDDs were unable to attenuate this linkage and may have attempted to downregulate their emotional response through maladaptive approaches, i.e., avoidance or suppression of the memory.

Greater hippocampal activity predicted subsequent increases in negative affect signature expression, during both memory recall and immerse trials for all subjects. These findings suggest that greater engagement of the hippocampus during memory recall and memory immersion may induce a stronger negative affective response. Hippocampal activity has previously been shown to be necessary for retrieval of specific details of memories^28^, however it was less clear how and whether hippocampal processes during encoding also include affective aspects of the memory. These findings demonstrate a direct link between hippocampal activity and negative affect during autobiographical memory retrieval. This work provides insights clinically, and also extends our understanding of the role of affect in the encoding and retrieval of autobiographical memories. Although the relationship between hippocampal activity and change in negative affect signature expression was similar for both groups during memory recall and immerse conditions, differences emerged during the distance condition.

Individuals with MDD differed from HVs in their neural approaches to downregulating negative affect. During the reappraisal condition, greater hippocampal activity predicted subsequent increases in negative affect signature expression in depressed individuals. In contrast, for HVs during the reappraisal condition, the relationship between hippocampal activity and change in negative affect signature expression was no longer present. HVs may have weakened the linkage between detailed memory retrieval and reactivation of affective details of those memories, and this may be due to the recruitment of a cognitive reappraisal neural network. During the distance condition in HVs, greater hippocampal activity predicted subsequent increases in emotion regulation signature expression. Increasing neural patterns that resemble an adaptative emotion regulation strategy, i.e., cognitive reappraisal, following memory engagement may have countered the effects of the memory reactivation on negative affect and explain why the relationship between hippocampal activity and negative affect was attenuated in HVs. In contrast, during reappraisal in MDDs, hippocampal activity predicted lower emotion regulation signature expression, which may explain the continued linkage between memory reactivation and negative affect. Interestingly, the relationship between hippocampal activity and negative affect signature expression was present only 1 TR (.9 seconds later) for both depressed individuals and HVs. Group differences emerged for both negative affect signature and emotion regulation signature expression when looking at change 2 TRs (1.8 seconds) later. This may be because the more immediate (i.e., less than 1 second) response to engaging with an upsetting memory is negative affect. However, 1–2 seconds later, HVs were able to engage compensatory emotion regulation strategies, which then attenuated the relationship between memory engagement and increased negative affect.

MDDs may disengage with a negative memory or suppress thoughts of the memory as a strategy for downregulating their negative affect. Our results indicate that individuals with MDD had a greater reduction in hippocampal activity compared with HVs when instructed to engage in cognitive reappraisal following the memory recall condition. Meta-analyses have shown that individuals with MDD employ less effective emotion regulation strategies (e.g., suppression, avoidance) and underuse effective strategies (e.g., cognitive reappraisal, acceptance)^4–6^. Some evidence suggests that depressed individuals use maladaptive strategies of suppression and avoidance when modulating emotion toward negative personal memories^2^. Our finding is consistent with prior neuroimaging work that showed downregulation of hippocampal-amygdala connectivity and hippocampal activity as a strategy for reducing negative affect in individuals with MDD but not HVs^16^. These findings suggest that the neural basis for this downregulation of negative affect in depressed individuals differs from HVs and may be maladaptive. When done correctly, cognitive reappraisal involves adjusting one’s interpretation or evaluation of a negative event while still holding that memory in mind^2, 5, 17, 18^. While disengaging with the negative memory may temporarily reduce negative emotions that the memory elicits, this is not an effective emotion regulation strategy long-term^2, 14^ and may reflect a broader impairment in emotion regulation that is characteristic of MDD.

### Limitations

One limitation is the study population was a predominately female sample (~ 70%), and future studies should strive for a more balanced gender distribution. Although we described the emotion regulation signature as a proxy for reappraisal, it is not necessarily a measure of effective emotion regulation. We did not examine hippocampal subfields, because not all subjects had a T2-weighted structural scan. Future work should explore the potential relationship of affect regulation to suicidal ideation and behavior. Inclusion of positive or neutral memories will provide an additional comparison of function in MDD and HVs.

### Conclusions

This study found neural differences in how depressed individuals regulate negative emotions to upsetting autobiographical memories. These differential patterns may reflect maladaptive reappraisal strategies. By harnessing advances in fMRI acquisition parameters and machine learning-based analysis techniques, we can better understand complex psychological processes in clinical populations and identify intervention targets for brain modulation therapies.

## Figures and Tables

**Figure 4:**
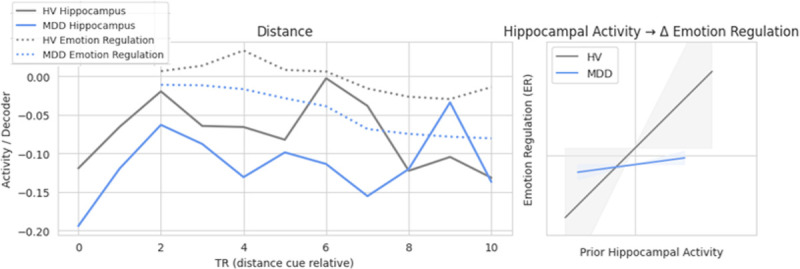
Temporal Relationship between Hippocampal Activity and Subsequent Emotion Regulation Signature (ERS) Expression During the Distance Condition Note: MDD: Major Depressive Disorder. HV: Healthy Volunteers. During the distance condition, there was an interaction of group by hippocampal activity on change in emotion regulation signature (ERS) expression 2 TRs later (p<.001). Greater hippocampal activity predicted subsequent increases in emotion regulation signature expression for HVs, but not for MDDs. There was no relationship between hippocampal activity and subsequent emotion regulation signature expression during the memory recall or immerse conditions in either group. Confidence intervals in the figure were approximated from simplified models due to random subject effects in full model.

**Table 1.** Demographic and Clinical Variables

Demographic and Clinical Variables	MDD (N=45)		HV (N=38)	
	Mean	SD (Range)	Mean	SD (Range)
**Age**	28.2	8.09 (19–55)	26.53	6.22 (18–51)
**Education, Years**	15.98	2.87 (12–27)	16.32	2.09 (12–23)
**BDI**	25.36	8.44 (8–42)		
	N	%	N	%
**Female**	32	71	27	71
**Race**				
**Asian**	12	27	11	29
**African American**	6	13	6	16
**Caucasian**	22	49	16	42
**Multiple**	5	11	5	13
**Hispanic**	11	24	9	24
**History of Suicide Attempt**	24	53		

Note: MDD: Major Depressive Disorder. HV: Healthy Volunteers. BDI: Beck Depression Inventory. There were no group differences in basic demographic characteristics (e.g., age, education, sex, ethnicity). The BDI is a measure of depression severity. The BDI is a 21-item self-report measure using a 4-point scale from 0–3, with higher scores indicating increased depression severity.
